# Association of atherogenic indices with C-reactive protein and risk factors to assess cardiovascular risk in rheumatoid arthritis patient at Tikur Anbessa Specialized Hospital, Addis Ababa

**DOI:** 10.1371/journal.pone.0269431

**Published:** 2022-06-03

**Authors:** Gashaw Dessie

**Affiliations:** Department of Biochemistry, School of Medicine, College of Medicine and Health Sciences, University of Gondar, Gondar, Ethiopia; Centro Cardiologico Monzino, ITALY

## Abstract

**Background:**

Rheumatoid arthritis (RA) is an autoimmune systemic chronic inflammatory disorder, which is characterized by joint stiffness, damage, and destruction of bone. In RA patients, the risk of cardiovascular disease is increased by 2–3 folds as compared to the general population. The major burden of RA is the development of cardiovascular diseases, including congestive heart failure, stroke, and myocardial infarction.

**Objectives:**

Assessment of the association of atherogenic indices with C-reactive protein to evaluate CVD risk was one of the purposes of this study. In addition, the association of atherogenic indices with elevated levels of cardiovascular risk factors (LDL-C and TG) was another aim of this study.

**Methods:**

The preferred study design for this study was a hospital based comparative cross-sectional study method. Data were cleaned, coded, and entered into Epi Data version 4.6 software, and exported to SPSS version 20 for further analysis of atherogenic indices, C-reactive protein, and risk factors. The comparison of atherogenic indices and other variables among the case and control groups was estimated by the independent t-test statistical analysis method. All variables with a p-value less than 0.2 during binary linear regression analysis were selected for multinomial logistic regression analysis. The association of atherogenic indices with C-reactive protein and risk factors was computed using multiple logistic regressions. The data were presented using tables and figures for clarification of the study.

**Results:**

The levels of atherogenic indices were computed for both RA patients and the control group. The values of atherogenic indices were significantly associated with cardiovascular risk factor (CRP ≥ 2mg/L). Atherogenic index of plasma (AIP) and TC/HDL-C ratio had a statistically significant association with an elevated levels of triglycerides (P<0.01). The TC/HDL-Cratio value of the patient had 2.38 folds more likely to have an elevated low density lipoprotein level. In addition, AIP of RA patients had 57.51 and 23.65 folds more to have elevated low density lipoprotein and triglycerides respectively.

**Conclusions:**

The result of this study showed that TC/HDL-C, LDL/HDL-C ratio values, and atherogenic index of plasma had a statistically significant association with elevated level of low density lipoprotein and triglycerides. In addition to this, they have a statistically significant association with the level of C-reactive protein. There was a highly significant statistical association between atherogenic indices, elevated low density lipoprotein, and triglycerides values. Therefore, the result of this finding confirmed that atherogenic indices have a potential role in the prediction and management of CVD risk in RA patients.

## Introduction

Rheumatoid arthritis (RA) is an autoimmune systemic chronic inflammatory disorder, which is characterized by joint stiffness, damage, and destruction of bone [1]. The risk of cardiovascular disease (CVD) is elevated by 2–3 folds among RA patients as compared to the general population [2]. In RA patients, cardiovascular disease is the predominant risk factor of mortality similar to CVD associated complications of diabetes mellitus. The congestive heart failure, stroke, ischaemic cardiomyopathy, and myocardial infarction are the common CVDs, which are mainly associated with the development of atherosclerosis [3, 4].

The atherogenic index of plasma (AIP), low density lipoprotein (LDL-C), high density lipoprotein (HDL-C), triglycerides (TG), and total cholesterol (TC) provide CVD risk prediction in RA patients [5, 6]. Of those lipid profiles, LDL-C and TG are potential biochemical markers to manage and assess cardiovascular risk estimation [7]. The assessment of CVD risk by atherogenic lipid profile is challenging due to the fluctuation effect of disease activity. However, atherogenic indices are more appropriate to assess CVD risk as they altered similar to the disease activity status [8]. An increase in atherogenic indices, including LDL/HDL-C and AIP correlated with a higher risk of CVD [9]. Although AIP is more preferable atherosclerotic biochemical parameter, TC/HDL-C, and LDL/HDL-C play a key role in CVD risk assessment compared with their measurement [10–12]. According to EULAR report, estimation of AIP is a good biochemical marker to assess and manage the risk of CVD in RA patients [7].

In RA patients, chronic inflammation activates dyslipidaemia, which is characterized by an elevated value of LDL-C and a decrease in HDL-C level [13]. The pathogenesis of RA is mainly associated with chronic inflammation, and it activates lipid profile derangement. Chronic inflammation is the major factor for complications of CVD risk in RA patients. Thus, the risk of CVD is independently associated with the inflammatory biochemical parameter, C-reactive protein [14]. The C-reactive protein (CRP) is correlated with atherosclerotic plaque formation and rupture, which in turn increases disease activity and risk of CVD [11, 15, 16]. Therefore, the risk of CVD is elevated during the inflammatory conditions, which is mainly correlated with the development of atherosclerosis [17].

The onset of chronic inflammation affects the level of HDL-C and triglycerides, which also alter atherogenic indices values [18]. The RA patient with CRP ≥ 2.6 mg/Lhad elevated atherogenic indices, which leads to the development of atherosclerosis [19]. The elevated ratio of TC/HDL-C is associated with an increased value of inflammatory marker (CRP) in RA patients. An increase in chronic inflammation leads to a reduction in the level of HDL-C, which in turn causes an increase in TC/HDL-C value [20]. On the other hand, the risk of CVD elevates in RA patients with smoking habit due to its association with severity and disease activity [18]. The mortality rate of CVD is more elevated in women RA patients than in men; thus, they are more affected by the disease [21]. Low socioeconomic status is correlated with high disease activity and risk of RA patients [22]. In Ethiopia, the potential role of atherogenic indices to assess the risk of CVD didn’t investigate among RA patients. In addition to this, the association of atherogenic indices with C-reactive protein and other associated risk factors didn’t well-defined. Therefore, the purpose of conducting the study was to assess the association of atherogenic indices with other cardiovascular risk factors and confirm their potential impact on the prediction and management of CVD risk in RA. In addition, the association of atherogenic indices with CRP to evaluate CVD risk was another aim of this study. On the other hand, the identification of demographic parameters correlating with CVD risk was also another objective of this study.

## Methods and materials

### Study area and period

The study was conducted at Tikur Anbessa Specialized Hospital rheumatology clinic. Tikur Anbessa Specialized Hospital is the largest referral teaching hospital in Ethiopia. It serves as the country’s top referral hospital. It has 800 beds and gives diagnostic and treatment services for about 370,000–400,000 patients per year. The study period of this study was from June 2018-November 2018.

### Study design method

The preferred study design for this study was a hospital based comparative cross-sectional study method. It was selected to evaluate the association atherogenic indices with C-reactive protein and other risk factors to assess CVD risk in RA patients and apparently healthy control groups.

### Source and study population

All rheumatoid patients who attend at the rheumatology clinic of Tikur Anbessa Specialized Hospital during the study period serve as a source population. Patients who meet the American College of Radiology (ACR) and European League Against Rheumatism (EULAR) RA criteria were categorized under the case group. In the same environmental setting, apparently healthy individuals who had no chronic diseases and willingness to provide signed informed consent were selected for control groups.

### Eligibility parameters of case and control groups

Rheumatoid arthritis (RA) patients who meet the American College of Radiology (ACR) and European League Against Rheumatism (EULAR) identification parameters were selected as the case groups. Patients with pregnancy, diabetes mellitus, hypertension, cancer, and liver disease were excluded. Additionally, patients with less than 18 years, and psychological disorders were excluded from the study. On the other hand, apparently healthy study participants who had no hypertension, diabetes mellitus, rheumatoid arthritis, pregnancy, cancer, and other chronic disease were included. In contrast to this, study participants who had a hearing and psychological problems, and less than 18 years old were excluded from the category of control group.

### Sample size determination and sampling technique

The size of study participants recruited into the research was calculated using single population proportion formula. The sample size was calculated by considering the level of confidence of 95%, and margin of error 5%. The proportion of occurrence of the events was considered with a similar context and proximate study setting. Due to the cost limitation of the study, the sizes of control groups were decreased as compared to the number of cases. Consequently, 73 RA patients and 40 apparently healthy control study participants were enrolled in this study.

### Sample and data collection methods

The data was collected from the study hospital within two months. The study was controlled and managed properly. The data collection process mainly focused on the objective of the study. On the other hand, the blood specimen was collected from both control and case groups after overnight fasting. Using BD Vacutainer EDTA coated test tube, 5ml venous blood was drawn and collected from both case and control groups. Whole blood was processed to centrifugation at 3000 revolutions per minute to distinguish serum. The separated serum was preserved at −20°C for appropriate laboratory investigation. Cobas Integra 400 Plus (Roche Diagnostics GmbH, Mannheim, Germany) clinical chemistry analyzer was utilized to analyze atherogenic lipid profile and CRP.

### Data processing and analysis

Data were cleaned, coded, and entered into Epi Data version 4.6 software, and exported to SPSS version 20 for further evaluation of atherogenic indices, CRP, and risk factors. The biochemical parameters and sociodemographic characteristics of study participants were analyzed using simple descriptive statistics. In addition, the data was presented using tables and figures for clarification of the study. The sociodemographic and clinical characteristics were compared among case and control groups using independent t-test. The association of indices with cardiovascular risk factors was computed using adjusted and odd ratio through binary and multiple linear regression analysis method. All variables with a p-value of less than 0.2 during binary linear regression analysis were selected for multivariable linear regression analysis. During analysis of the study, adjusted, crude odd ratio, and 95% CI was computed. P-value < 0.05 was considered as statistically significant.

### Ethical approval and informed consent

The ethics committee of the Biochemistry department of Addis Ababa University approves and decided on the protocol. The research and ethics committee approve it by reference number, SOM/ BCHM/137/2010 on 11/06/2018. Written informed consent was provided to all of the study participants and it is available from the corresponding author.

## Results

### Comparison of atherogenic indices and biochemical parameters among study participants

The levels of atherogenic indices (TC/HDL-C, LDL/HDL/L, and AIP) were computed for both RA patients and control groups. In RA patients, the Mean ± SD value of AIP, TC/HDL, and LDL/HDL were 0.36 ± 0.26, 3.64 ± 1.52, and 2.07 ±1.18 respectively. On the other hand, the Mean ± SD value of AIP, TC/HDL, and LDL/HDL for apparently healthy study participants were 0.28 ± 0.19, 3.40 ± 0.84, and 1.98 ± 0.73 respectively. The mean ± SD of hsCRP were 10.54±17.26 and 3.54 ±7.60 for both case and control groups respectively.

### Atherogenic index of plasma, hsCRP and lipid profile category of study participants

The atherogenic lipid profile and indices were estimated for both RA patients and control groups.

Regarding TG value, 16 (21.91), 7 (9.58), and 50 (68.49) of RA patients were categorized under≥175 mg/L, 150-175mg/L and < 150mg/L respectively. Of the total control group members, 2, 2, and 36 of them had ≥175 mg/L, 150-175mg/L, and <150mg/L triglyceride (TG) values respectively. Additionally, 53 (72.60), 10 (13.69), and10 (13.69) patients had higher, medium, and lower AIP values respectively. In contrast, 22 (55), 11 (27.5) and 7 (17.5) apparently healthy study participants had higher, medium, and lower AIP values respectively.

### Evaluation of association of atherogenic indices, hsCRP and demographic data with CVD risk factors

According to 2018 American College of Cardiology/American Heart Association clinical practice guidelines, lipid profile estimation with TG ≥175mg/L and LDL >160mg/L were regarded as a risk enhancer (factor) of CVD. The TC/HDL value of the patient had 2.38 and 3.47 times more likely to have elevated LDL-C and TG levels respectively. Similarly, AIP of RA patients had 57.51 and 23.65 folds more to have elevated LDL-C and TG respectively. Patients with ≥ 2mg/L level of hsCRP had 3.15 and 7.58 times more likely to have elevated LDL-C and TG values than patients having <2mg/L. Patients with < 40mg/dl HDL value had 1.99 and 4.55 times more likely to have elevated LDL-C and TG values respectively.

### Association of atherogenic indices and demographic data with hsCRP

Rheumatoid arthritis patients with >0.21 AIP value were 4.99 times more likely to have elevated hsCRP levels as compared to patients having<0.21 value. Similarly, patients with ≥175mg/L TG values had 7.58 times more likely to have a higher value of hsCRP as compared to patients with < 150mg/L. The values of atherogenic indices, including TC/HDL-C, LDL/HDL-C, and Log (TG/HDL-C) were significantly associated with an increase in the level of hsCRP. On the other hand, patients with low income had 12.07 times more likely to have elevated cardiovascular risk factor as compared to patients having higher income status.

### Comparison of atherogenic index of plasma regarding hsCRP level

From the total RA patients, 36, 3, and 3 study participants were categorized under >0.21, 0.11–0.21 and < 0.11 AIP values respectively. Of the total RA patients, 36 of them had elevated AIP (>0.21) and hsCRP (≥2mg/L) levels. In contrast, 7 of them had lower hsCRP and AIP values. Thus, 42 patients had elevated hsCRP measurement.

### Triglyceride category evaluation regarding hsCRP level of RA patients

Of the total RA patients, 14 of them had both elevated levels of TG and hsCRP values. In contrast, 26 of them were categorized under lower hsCRP and TG values. Thus, 42 patients had elevated hsCRP values. On the other hand, 50 of them had a lower level of TG.

## Discussion

The result of this study showed that the atherogenic indices are the potential biochemical markers to predict and manage the risk of cardiovascular disease in RA patients. In this study, the atherogenic indices, including AIP, TC/HDL-C, and LDL/HDL-Chad a statistically significant association with well-defined cardiovascular risk factors. The assessment of this study showed that women were more likely to have elevated LDL-C and TG values than men (Table 3). Thus, it was agreed with the previous finding [1]. On the other hand, the Mean ± SD of BMI value showed that RA patients had a higher level as compared to control. However, it didn’t show a statistically significant variation. The previous investigation also reports about the lack of significant association of BMI value among patients and apparently healthy study participants [23]. Regarding the assessment of socioeconomic status, RA patients with low monthly income had more likely to have elevated cardiovascular risk factors (Table 4). Low income status of study participants was significantly associated with hsCRP, and it was agreed with the study done in Turk [24].

The result of this study showed that atherogenic indices, including AIP, TC/HDL-C, and LDL/HDL-C had no a statistically significant difference in both case and control groups. It may be due to the effect of lipid-lowering agents in RA patients. The result was consistent with another study [25]. However, there was a relative elevation of indices among patients than apparently healthy control group. It was in line with previous investigation done in Iran, which revealed that both LDL/HDL-C and TC/HDL-C were elevated among RA patients as compared to the control group [26]. On the other hand, the value of hsCRP showed a statistically significant elevation among patients as compared to controls (Table 1). It may be due to chronic inflammation, which in turn leads to lipid metabolism derangement [13]. During the assessment of this study, AIP was also calculated by log (TG/HDL-C). The majority of RA patients had elevated AIP levels (Table 2). The result of this study was in line with another finding [6]. Similarly, the level of categorical values of the atherogenic lipid profile and hsCRP were higher among patients than the control group.

**Table 1:** Comparison of atherogenic indices and hsCRP among case and control group.

Characteristics	Case (n = 73)	Control (n = 40)	P-value
LDL/HDL	2.07 ±1.18	1.98 ± 0.73	0.65
TC/HDL	3.64 ± 1.52	3.40 ± 0.84	0.36
AIP	0.36 ± 0.26	0.28 ± 0.19	0.11
hsCRP	10.54 ± 17.26	3.54 ± 7.60	0.01*
BMI	23.25 ± 3.67	23.87 ± 3.77	0.40

(AIP; Atherogenic index of plasma, BMI; Body mass index, HDL; high density lipoprotein, hsCRP; high sensitivity C-reactive protein, LDL; low density lipoprotein, TC; total cholesterol; * indicates P<0.05).

**Table 2:** Comparison of categorical value atherogenic lipid profile among study participants.

Characteristics	Category	Cases n (%) (n = 73)	Controls n (%) (n = 40)
AIP	>0.21	53 (72.60)	22 (55)
	0.11–0.21	10 (13.69)	11 (27.5)
	< 0.11	10 (13.69)	7 (17.5)
LDL-C	160-189mg/L	5 (6.84)	1 (2.50)
	< 160mg/L	68 (93.15)	39 (97.50)
TG	≥ 175 mg/L	16 (21.91)	2 (5.00)
	150-175mg/L	7 (9.58)	2 (5.00)
	< 150mg/L	50 (68.49)	36 (90.0)
HDL-C	< 40mg/L	18 (24.65)	10 (25.00)
	≥ 40mg/L	55 (75.34)	30 (75.00)
hsCRP	≥ 2mg/L	42 (57.53)	7 (17.50)
	˂ 2mg/L	31 (42.46)	33 (82.50)

(AIP; Atherogenic index of plasma, BMI; Body mass index, HDL; high density lipoprotein, hsCRP; high sensitivity C-reactive protein, LDL; low density lipoprotein, mg/L; milligrams per liter, n; number of study participants, TC; total cholesterol; * indicates P<0.05).

**Fig 1:**
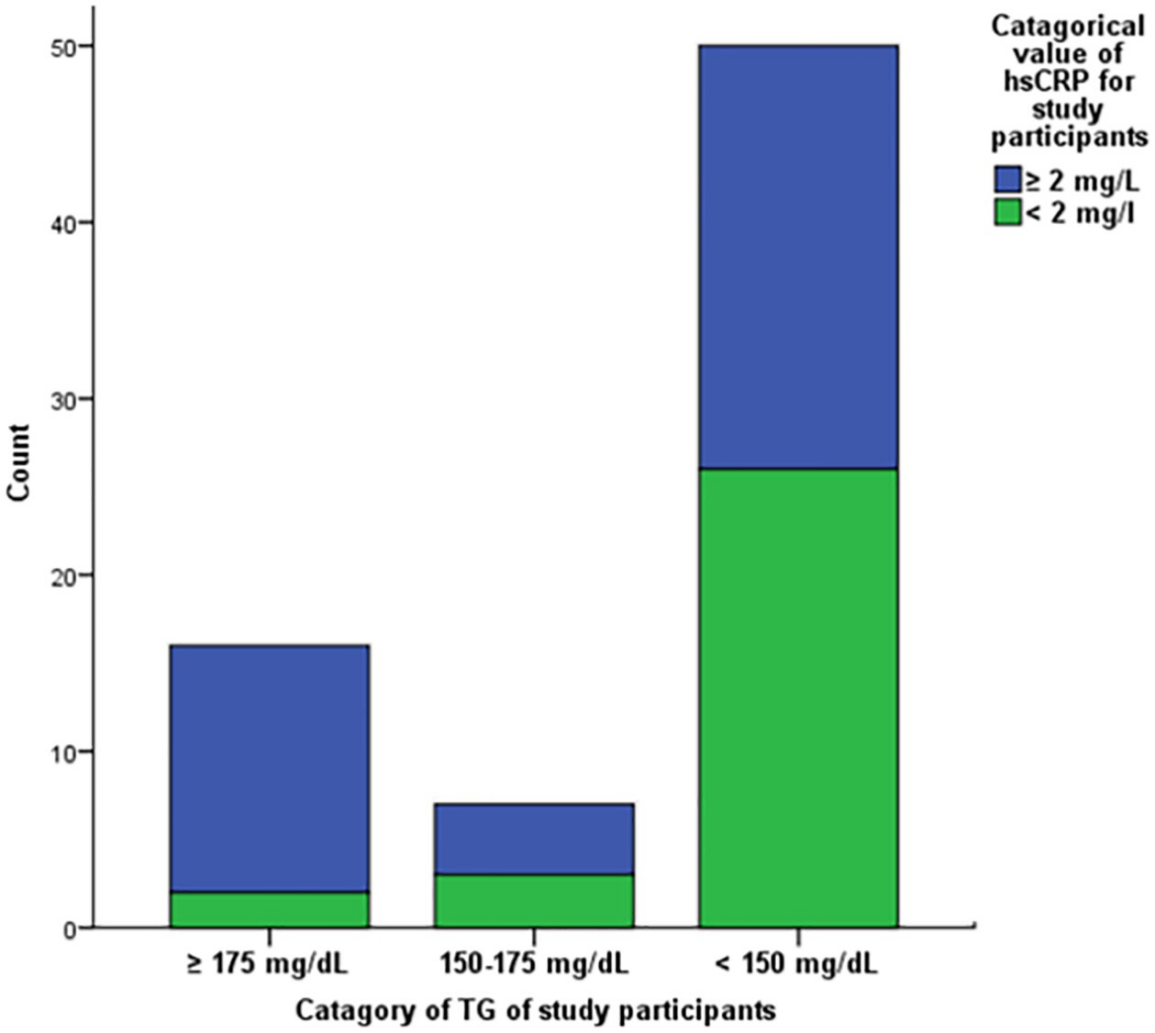
Comparison of triglyceride category with hsCRP of RA patients.

**Fig 2:**
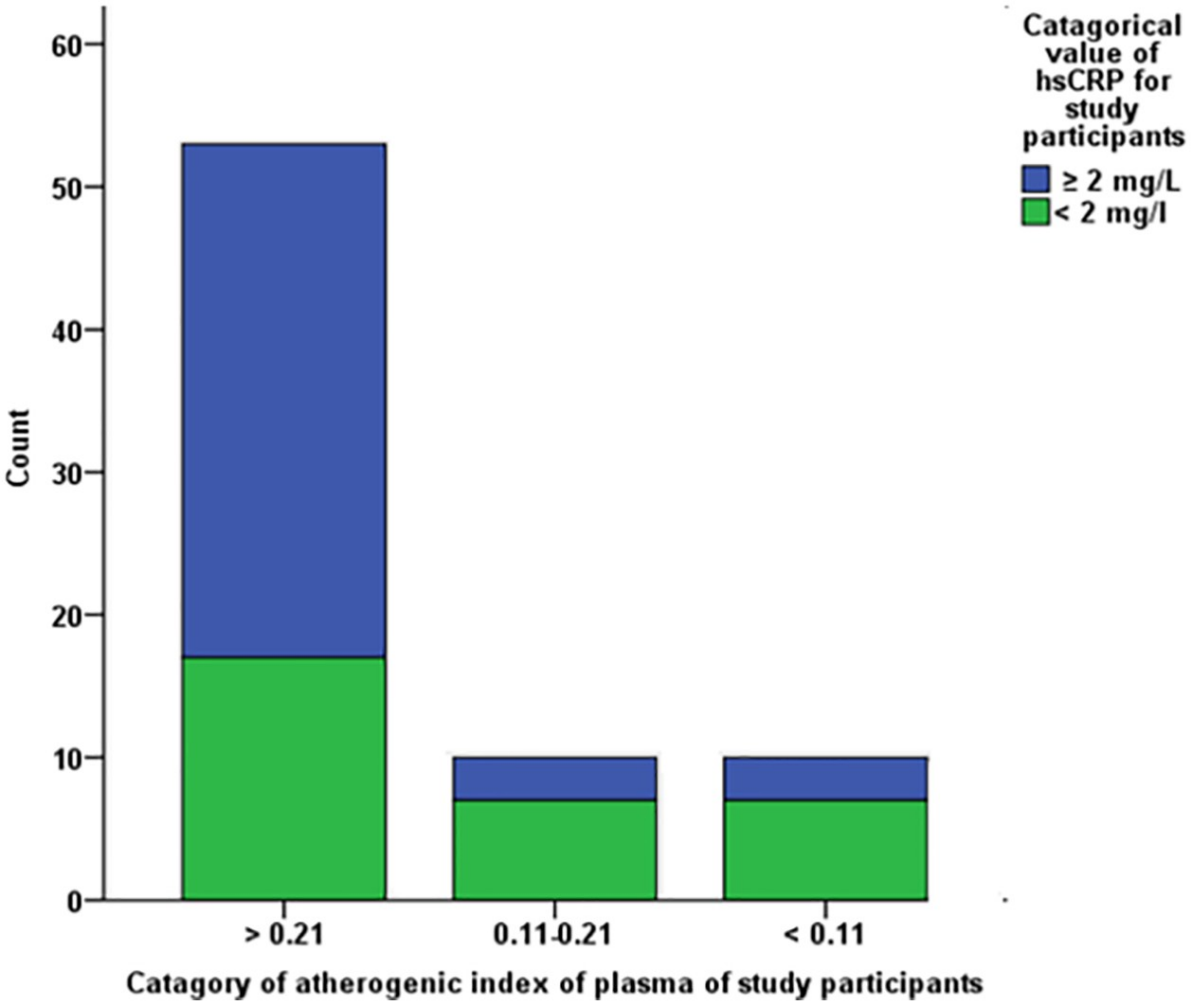
Comparison of atherogenic index of plasma with hsCRP of RA patient.

On the other hand, the association between atherogenic indices and inflammatory marker were evaluated in this study. The 2018 American College of Cardiology/American Heart Association report revealed that cardiovascular risk in RA patients is associated with elevated LDL-C, TG, and hsCRP levels. This clinical practice guideline explained that RA patients with TG≥175mg/L, LDL>160mg/L, and hsCRP ≥ 2mg/L had elevated CVD risk [27]. Of the total RA patients, the majority of them (57.53%) had elevated hsCRP levels (Table 2 and Fig 1). In addition, the majority of RA patients (49.31%) showed an increase in the level of AIP (Fig 2). In this study, elevation of AIP, TG, and LDL-C was reported in line with the previous case study investigation done in Chicago, U.S.A. [28].

The level of TC/HDL-C and AIP showed a statistically significant association with elevated values of LDL-C. Patients with elevated AIP levels were more likely to have elevated LDL-C and TG values. It was agreed with the previous finding [29]. The result of this study showed that TC/HDL-C ratio had a statistically significant association with those cardiovascular risk factors (Table 3). Therefore, the level of AIP and TC/HDL-C become elevated as both risk factors increased. The previous investigations done in Japan showed that TC/HDL-C ratio becomes elevated among patients with triglyceridaemia and high disease activity. The result of this current study was agreed with another previous investigation [30]. In contrast, the other concise report done in the United Kingdom showed that there was no alteration in the level of TC/HDL-C ratio during the assessment of association [31]. RA patients with reduced HDL-C levels were more likely to have elevated TG values (Table 3).

**Table 3:** Multiple logistic regression of atherogenic indices and demographic data with cardiovascular risk factors.

Variables	Category	CVD risk factor (LDL >160mg/L) (n = 73)				CVD risk factor (TG ≥ 175mg/L) (n = 73)			
		AOR	COR	95% CI	P-value	AOR	COR	95%CI	P-value
HDL	< 40mg/L	1.99	0.68	0.279–14.22	0.49	4.55	1.51	1.34–15.38	0.01*
	≥ 40mg/L	1	1			1	1		
TC/HDL	-	2.38	0.86	1.35–4.17	0.003*	3.47	1.24	1.86–6.47	0.00*
AIP	-	57.51	4.05	1.66–19.90	0.02*	23.65	14.67	17.59–31.80	0.00*
Sex	Female	0.53	0.62	0.53–5.83	0.59	0.48	0.73	0.10–2.28	0.35
	Male	1	1			1	1		
hsCRP	≥2mg/L	3.15	1.15	0.33–29.75	0.31	7.58	2.02	1.55–36.89	0.01*
	<2mg/L	1	1			1	1		
Smoking	Yes	5.41	1.68	0.45–64.56	0.18	3.42	1.23	0.44–26.59	0.23
	No	1	1			1	1		
RF	Reactive	0.46	0.77	0.071–3.00	0.41	0.55	0.59	0.15–1.94	0.35
	NR	1	1			1	1		
BMI	> 25	0.69	0.36	0.07–6.62	0.74	6.00	1.79	0.49–72.20	0.15
	25.9–29.9	1	1	-	0.46	0.46	0.77	0.09–2.34	0.35
	<25	1	1			1	1		

(AOR; Adjusted odd ratio, CVD; cardiovascular disease, CI; confidence interval, COR; crude odd ratio, n; number of patients, TG; triglyceride).

On the other hand, patients with an increased hsCRP level had more likely to have elevated CVD risk as compared to patients with decreased hsCRP values (Table 3). The previous investigations done in India showed that the ratio of TC/HDL-C was elevated in patients, and they confirmed the presence of an association between hsCRP and TC/HDL-C [32]. Consequently, it was in line with the result of this study. On the other hand, patients with elevated AIP levels had more likely to have elevated hsCRP (Table 4). Similar to the result of this study, research done in India showed that AIP was associated with hsCRP and the risk of CVD [5]. Additionally, RA patients with increased LDL/HDL-C ratio were more likely to have a higher level of hsCRP. In contrast to this, a recent study reported that RA patients with an increase in the level of hsCRP had decreased value of LDL-C [33]. But, the study didn’t mention their correlation and CVD. On the other hand, the level of TG was significantly associated with high disease activity status.

**Table 4:** Comparison of association of demographic and biochemical parameters with hsCRP.

Variables	CVD risk factor (hsCRP ≥ 2mg/l) (n = 73)				
	Values	AOR	COR	95% CI	P-value
HDL	<40mg/L	3.37	1.21	0.98–11.55	0.05
	≥ 40mg/L	1	1		
AIP	>0.21	4.94	1.59	1.13–21.49	0.03*
	<0.11	1	1		
LDL	160-189mg/l	3.15	1.15	0.33–29.75	0.31
	<160mg/l	1	1		
TG	≥175mg/L	7.58	2.02	1.55–36.89	0.01*
	150-175mg/L	1.44	0.36	0.29–7.12	0.65
	<150mg/L	1	1		
TC/HDL	-	1.83	0.60	1.14–2.92	0.01*
LDL/HDL	-	1.95	0.67	1.12–3.39	0.01*
Log (TG/HDL)	-	48.73	3.886	4.01–59.14	0.002*
Sex	Female	0.64	0.44	0.14–2.79	0.55
	Male	1	1		
Monthly income	<500 ETB	12.07	2.49	2.98–48.81	0.00*
	500–1000 ETB	3.54	1.26	0.88–14.19	0.07
	>1000 ETB	1	1		
Educational level	Illiterate	1.224	0.20	0.27–5.37	0.78
	Primary school	1.03	0.03	0.24–4.30	0.96
	Secondary school	0.71	0.336	0.16–3.02	0.64
	Higher education	1	1		

(AOR; Adjusted odd ratio, CVD; cardiovascular disease, CI; confidence interval, COR; crude odd ratio, n; number of patients, TG; triglyceride).

## Conclusions

In this study, the level of atherogenic indices didn’t show a statistically significant difference between RA patients and control groups. The result of this study showed that TC/HDL-C, LDL/HDL-C, and AIP had a statistically significant association with an elevated level of LDL-C and TG, which are well-defined cardiovascular risk factors. Similarly, they have a statistically significant association with hsCRP, which is another known cardiovascular risk factor and disease activity biochemical marker. There was a highly significant statistical association between atherogenic indices, elevated LDL-C, and TG values. The association of indices with cardiovascular risk factors showed that atherogenic indices may act as accurate markers to assess CVD risk due to fluctuation effect of disease activity. Therefore, the CVD risk assessment may be more enhanced by evaluation of atherogenic indices as compared to individual lipid profiles. Consequently, the result of this finding confirmed that atherogenic indices have the potential role in the prediction and management of CVD risk in RA patients.

## Abbreviations

AIP: Atherogenic index of plasma
BMI: body mass index
HDL-C: high density lipoprotein
hsCRP: high sensitivity C-reactive proteins
LDL-C: low density lipoprotein
RA: Rheumatoid arthritis
TC: total cholesterol
TG: triglycerides
